# Comparison of the editing patterns and editing efficiencies of TALEN and CRISPR-Cas9 when targeting the human CCR5 gene

**DOI:** 10.1590/1678-4685-GMB-2017-0065

**Published:** 2018-03-19

**Authors:** Arildo Nerys-Junior, Luciene P. Braga-Dias, Paula Pezzuto, Vinícius Cotta-de-Almeida, Amilcar Tanuri

**Affiliations:** 1 Laboratório de Virologia Molecular, Universidade Federal do Rio de Janeiro (UFRJ), Rio de Janeiro, RJ, Brazil; 2 Laboratório do Serviço de Biotecnologia e Desenvolvimento Animal, Instituto de Ciência e Tecnologia em Biomodelos, Fundação Oswaldo Cruz, Rio de Janeiro, RJ, Brazil; 3 Laboratório de Pesquisas Sobre o Timo, Fundação Oswaldo Cruz, Rio de Janeiro, RJ, Brazil

**Keywords:** CCR5, CRISPR-Cas9, efficiency, gene editing, TALEN

## Abstract

The human C-C chemokine receptor type-5 (CCR5) is the major transmembrane co-receptor that mediates HIV-1 entry into target CD4+ cells. Gene therapy to knock-out the CCR5 gene has shown encouraging results in providing a functional cure for HIV-1 infection. In gene therapy strategies, the initial region of the CCR5 gene is a hotspot for producing functional gene knock-out. Such target gene editing can be done using programmable endonucleases such as transcription activator-like effector nucleases (TALEN) or clustered regularly interspaced short palindromic repeats (CRISPR-Cas9). These two gene editing approaches are the most modern and effective tools for precise gene modification. However, little is known of potential differences in the efficiencies of TALEN and CRISPR-Cas9 for editing the beginning of the CCR5 gene. To examine which of these two methods is best for gene therapy, we compared the patterns and amount of editing at the beginning of the CCR5 gene using TALEN and CRISPR-Cas9 followed by DNA sequencing. This comparison revealed that CRISPR-Cas9 mediated the sorting of cells that contained 4.8 times more gene editing than TALEN+ transfected cells.

## Introduction

HIV-1 entry into target CD4+ cells requires the C-C chemokine receptor type 5 (CCR5) that acts as a co-receptor for the V3 loop of the gp120 viral adhesion protein (Hill *et al.*, 1997). In addition, mutant strains raised in advanced stages of the infection can use the C-X-C chemokine receptor type 4 (CXCR4) as a co-receptor to mediate viral entry. However, CXCR4 has not been defined as a preferential anti-HIV target since it is also the transmembrane protein that guides CD4+ cells to inflammatory sites and is thus regarded as highly relevant for immune activity (McGowan and Shah, 2010). On the other hand, a CCR5 natural 32-bp deletion (defined as the CCR5Δ32 allele) is an effective restriction condition against HIV-1 infection. As this mutant allele produces a truncated protein that is not expressed on the cell surface, individuals homozygous for CCR5Δ32 cannot be infected by the usual CCR5-tropic-only strains of HIV-1 (Grotto and Pardini, 2006). This mutation thus confers resistance to HIV-1 infection in the homozygous state and partial resistance to infection with a slower rate of progression to AIDS in the heterozygous state (Reiche *et al.*, 2008; Silva-Carvalho *et al.*, 2016). In addition, this mutant allele has been associated with increased susceptibility to systemic lupus erythematous (Baltus *et al.*, 2016) and juvenile idiopathic arthritis (Scheibel *et al.*, 2008), as well as decreased susceptibility to pre-eclampsia (Telini *et al.*, 2014), osteomyelitis (Souza *et al.*, 2015) and rheumatoid arthritis (Pokorny *et al.*, 2005).

In a landmark heterologous transplant in 2009, an HIV-1-positive patient received a bone marrow transplant from a compatible HIV-1-negative CCR5Δ32 homozygote donor as treatment for his acute myeloid leukemia (Hütter *et al.*, 2009). After transplantation, antiretroviral therapy was discontinued, resulting in a rapid decrease in viral load followed by long-term viral absence. This was considered the first functional cure for HIV-1 infection, with the patient remaining functionally healed.

Despite the very encouraging results of heterologous transplantation, the large-scale application of this approach is not a trivial matter, mainly because of the low frequency of the CCR5Δ32 allele in the general population (10-20% in northern and northeastern Europe, which have the highest frequencies of CCR5Δ32 in the world) and the low frequency of compatible individuals (Gonzales *et al.*, 2001; Hütter *et al.*, 2009; Silva-Carvalho *et al.*, 2016).

To overcome these problems, gene therapy strategies in autologous transplantation have been proposed to treat HIV-1 infection (Hütter *et al.*, 2009; Cannon and June, 2011). In addition, the beginning of the CCR5 gene, defined as the 3’ nucleotides immediately downstream from the ATG start codon, is a key region for planned targeting since it mediates properly CCR5 gene knock-out. Gene therapy for HIV-1 infection initially requires the identification and choice of a suitable genetic tool for editing the target gene. Currently, the two most modern and effective programmable endonucleases that mediate precise gene targeting are the transcription activator-like effector nucleases (TALEN) and clustered regularly interspaced short palindromic repeats (CRISPR-Cas9) (Nemudryi *et al.*, 2014).

Chromosomal position is directly related to chromatin structure, and transcriptional rate (Narlikar *et al.*, 2002), as well as promoter and genetic position are directly related to epigenetic modifications such as DNA methylation (Moarii *et al.*, 2015). TALEN binds to methylated cytosines (Valton *et al.*, 2012a,b), whereas CRISPR-Cas9 does not (Vojta *et al.*, 2016). However, additional studies are needed to determine the patterns of sensitivity for TALEN and CRISPR-Cas9 in chromatin, epigenetics, histones, nuclear localization and different transcriptional landscapes. To determine the relevance of these conditions in targeting the genetic site prior to gene editing it is very desirable to understand the patterns and efficiency of gene editing using TALEN and CRISPR-Cas9 for each genetic region of interest in the target cell type (Arvey *et al.*, 2012; Lee *et al.*, 2012; Weber *et al.*, 2015).

TALEN and CRISPR-Cas9 are endonucleases that can be programmed to target DNA cleavage. TALEN recognizes thymine on it’s conserved n-terminal portion. This is the position “0" (zero) of the genomic target. Subsequent target genomic recognition process is performed by the assembled sequence of specific repetitive variable diresidue (RVD), where: ”NI" RVD type recognizes adenine, “HD” RVD type recognizes cytosine, “NN” RVD type recognizes guanine and “NG” RVD type recognizes thymine; other forms allow additional nucleotide recognition with lower efficiency and double-strand breaks can be produced by dimerization of the *Fok*I catalytic site from both TALEN arms (Cermak *et al.*, 2011a). In contrast, CRISPR recognizes the target through a short RNA sequence known as single guided RNA (sgRNA) and double-strand breaks are produced by the Cas9 protein from adaptive bacterial immunity Type II of *Streptococcus* spp., e.g., *Streptococcus pyogenes*, and *Archaeae* (Gasiunas *et al.*, 2012; Jinek *et al.*, 2012; Wiedenheft *et al.*, 2012; Riordan *et al.*, 2015).

The CRISPR-Cas9 target requires a protospacer adjacent motif (PAM) sequence (5’-NGG-3’) and sgRNA has an anchorage sequence that anchors it to Cas9. This anchorage sequence is followed by the recognition sequence (without a PAM complementary sequence) that is complementary to the target. A double-strand break occurs in the base pairs after the PAM sequence (three base pairs after the beginning of the recognition sequence) where the HNH domain of Cas9 cleaves the strand that is paired with sgRNA and the RuvC domain cleaves the other DNA strand at the same position (Jinek *et al.*, 2012; Wiedenheft *et al.*, 2012; Riordan *et al.*, 2015). Whereas TALENs are assembled by a sequence of cloning and subcloning steps (Cermak *et al.*, 2011b), CRISPR-Cas9 is easily assembled in a single cloning step (Ran *et al.*, 2013a). This difference makes TALEN more time consuming to assemble compared to CRISPR-Cas9.

TALEN requires a reporter plasmid to indicate its action within the cell (Kim *et al.*, 2011). This plasmid has an operon modulated by a CMV promoter that regulates the expression of a red fluorescent protein (RFP) which, in turn, indicates successful transfection of the plasmid. This RFP sequence is immediately followed (without a stop codon) by the TALEN recognition sequence and an out-of-frame GFP sequence. Whenever TALEN is expressed and cleaves the plasmidial target, the non-homologous end-joining cellular repair mechanism inserts an InDel mutation that reestablishes the GFP frame in some cases (Kim *et al.*, 2011). For this to occur, the TALEN target must not contain a stop codon in the frame containing GFP. In some cases, TALEN cleaves the target but does not reestablish the GFP open reading frame. Conversely, several CRISPR-Cas9-coding plasmids already have a GFP reporter gene after the Cas9 gene where it is separated from the Cas9 protein by a T2A self-cleaving peptide (Ran *et al.*, 2013a). TALEN transfections require the co-transfection of three plasmids at the same time (right arm plasmid, left arm plasmid and reporter plasmid), but the reporter plasmid is optional in some cases (Kim *et al.*, 2011). On the other hand, CRISPR-Cas9 transfections require the transfection of a single plasmid that contains not only all the CRISPR-Cas9 molecular requirements to cleave the desired target, but also a puromycin resistance gene for drug-based cell selection, or a GFP reporter system that indicates Cas9 production by itself (Ran *et al.*, 2013a).

Cell sorting can be done in both TALEN and CRISPR transfection experiments, although in TALEN transfections, the use of a reporter plasmid to sort RFP^+^/GFP^+^ cells (produced by TALEN when acting in the nucleus) is required, (Kim *et al.*, 2011). In contrast, CRISPR-Cas9 transfections may allow the sorting of GFP^+^ cells by themselves, where GFP^+^ cells are produced by Cas9-T2A-GFP Open Reading Frame (ORF) before Cas9 anchoring in the sgRNA produced by the same plasmid (Ran *et al.*, 2013a).

TALEN has low toxicity and is very efficient, very specific and rarely shows off-target effects (Mussolino *et al.*, 2011). In contrast, CRISPR-Cas9, despite being more efficient than TALEN, may generate higher off-target effects (Shen *et al.*, 2014; Tsai and Joung, 2016). These off-target effects of CRISPR-Cas9 can be easily and dramatically reduced by using truncated single-guided RNAs (sgRNAs < 20 nucleotides in length) (Fu *et al.*, 2014).

CRISPR-Cas 9 is highly efficient at inducing mutagenesis in certain human somatic cells and this characteristic can be used to mediate hematopoietic cell-based therapy (Mandal *et al.*, 2014). However, the differences in efficiency between CRISPR-Cas9 and TALEN should be tested for each target locus in each target cell type to assess the usefulness of these tools for each objective. This applies to cell cultures such as HEK293T cells and genes, such as the human CCR5 gene, that are targets for gene therapy.

The therapeutic potential of CRISPR-Cas9 has been known and explored for some time (Gaj *et al.*, 2013). Although CRISPR-Cas9 is better than TALEN for gene editing in HEK293FT cells (He *et al.*, 2016), it is unclear to what extent CRISPR-Cas9 is better than TALEN at editing the human CCR5 gene, including in HEK293T cells.

Gene therapy for the human CCR5 gene is ever closer to becoming a reality, and CRISPR-Cas9 has a prominent role in this process. The ablation of the CCR5 gene in NOD/Prkdc^scid^/IL-2Rγ^null^ mice was found to confer long-term resistance to HIV-1 infection *in vivo* (Xu *et al.*, 2017). This finding has renewed interest in gene-therapy-based alternatives for curing HIV-1 infections using a hematopoietic stem cell procedure. The *in vivo* excision of HIV-1 provirus with a multiplex CRISPR-Cas9 system has been used other animal models, such as transgenic mice, and may provide an alternative approach for gene therapy through CRISPR-Cas9 (Yin *et al.*, 2017).

Despite various advances in the use of gene therapy to treat HIV-1 infection, and the fact that TALEN and CRISPR-Cas9 have been used in most studies, including targeting of the CCR5 gene, nothing is known about possible variations in the patterns and efficiencies of TALEN and CRISPR-Cas9 in editing the beginning of the CCR5 gene. Such knowledge is important for corroborating the choice of a given tool in basic research and gene therapy targeting of the CCR5 gene.

To address our poor understanding of this matter, and to determine whether TALEN or CRISPR-Cas9 is the better of these two approaches for editing the beginning of the CCR5 gene, we compared the patterns and quantity of editions in the CCR5 gene using TALEN and CRISPR-Cas9. DNA sequencing was used to show that a version of CRISPR-Cas9 that carries a GFP reporter gene mediates the sorting of cells that contain five times more gene editing than the sorted TALEN^+^ transfected cells.

## Material and Methods

### TALEN assembly and reporter plasmid construction

**Figure 1:**
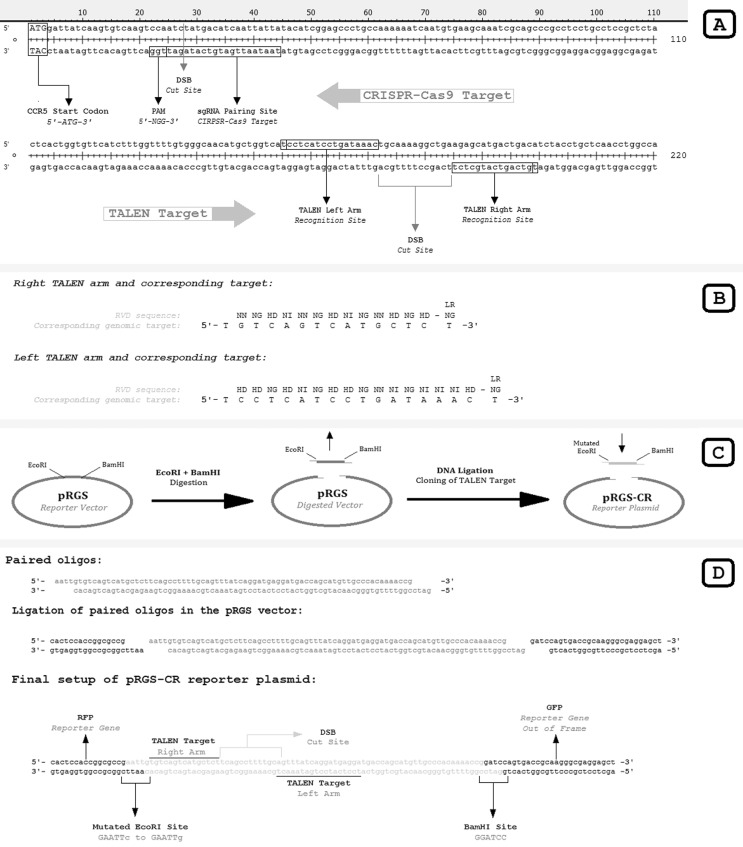
CRISPR-Cas9 and TALEN recognition sites with assembly description and pRGS-CR reporter plasmid construction. **A.** The beginning of the CCR5 gene with TALEN and CRISPR-Cas9 recognition sites. Whereas the assembled CRISPR-Cas9 mediates a blunt double-strand break between the 24^th^ and 25^th^ nucleotides from the CCR5 start codon (ATG), TALEN mediates an overhanging double-strand break (DSB) between the 168^th^ and 181^th^ nucleotides from the CCR5 start codon. **B.** Repetitive variable diresidue (RVD) sequence of right and left TALEN arms with corresponding genomic recognition site. LR indicates the last repeat RVD. **C.** Steps of pRGS-CR reporter plasmid assembly. The pRGS vector (Plasmidial Red and Green system) is co-digested with *Eco*RI and *Bam*HI to release a short DNA strand and expose *Eco*RI and *Bam*HI overhangs. Annealed oligos are inserted in the digested pRGS vector through corresponding overhangs. The annealed oligos contain a mutated *Eco*RI site 5’-GAATTc-3’ (right) to 5’-GAATTg-3’ (mutated) in the *Eco*RI overhang to allow digestion before transformation, thereby avoiding the transformation of unwanted assembled constructs. **D.** Details of the assembled pRGS-CR reporter plasmid. Annealed oligos with corresponding overhangs are indicated, as are the recognition sites for each TALEN arm, the *Bam*HI splicing site and the mutated *Eco*RI sequence of the annealed oligos.

To target the beginning of the CCR5 gene, the TALEN strategy proposed by Miller *et al.* (2011) was assembled through the Golden Gate TALEN assembly kit (AddGene, Cambridge, MA, USA) (Cermak *et al.*, 2011b). For gene editing, both right and left assembled TALEN plasmids were transfected together, with the recognition site starting 154 bp downstream from the CCR5 start codon (ATG) relative to the first standard thymine (T) of the recognition site. To demonstrate TALEN activity within cells (Kim *et al.*, 2011), a reporter plasmid containing the TALEN target was constructed using the *pRGS* vector (red-green system plasmid), referred to as the pRGS-CR reporter plasmid (pRGS to CCR5 Miller TALEN target) (Figure 1).

### CRISPR-Cas9 assembly

**Figure 2:**
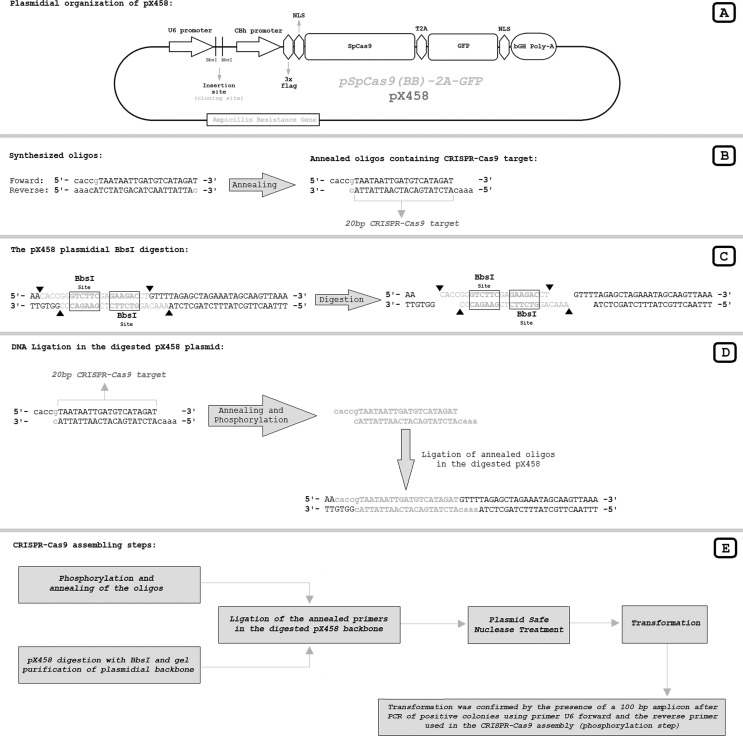
Sequential steps for assembling the CRISPR-Cas9 plasmid using the pX458 vector. **A.** The pX458 vector (also known as pSpCas9(BB)-2A-GFP; Addgene Plasmid #48138) map. The U6 promoter encodes short RNAs and regulates the synthesis of sgRNA. The CBh promoter modulates Cas9 expression that is followed by a T2A self-cleaving peptide that releases a GFP reporter protein. The bovine growth hormone polyadenylation signal (bGH-PolyA) is used after the GFP gene. **B.** Oligos used to assemble the CRISPR-Cas9. Annealed oligos have overhangs complementary to the *Bbs*I digested pX458 vector. The 20-bp genomic CRISPR-Cas9 target that encodes the genomic recognition part of sgRNA is indicated. The gray highlighted guanine (g) that is paired with the gray highlighted cytosine (c) are not part of the recognition site of sgRNA, but are requirements of the U6 promoter for proper sgRNA production. **C.** Details of *Bbs*I digestion of the pX458 vector. Both *Bbs*I sites are released in the digestion since they are located within the DNA sequence that is replaced by the annealed oligos in the ligation. **D.** Detailed ligation of annealed oligos in the *Bbs*I-digested pX458 vector. **E.** General steps for CRISPR-Cas9 assembly.

The beginning of the CCR5 gene was analyzed using the online software CRISPR design tool from the Massachusetts Institute of Technology (available at http://crispr.mit.edu/) (Hsu *et al.*, 2013). The nearest possible CRISPR target site from the CCR5 start codon was chosen for testing (Figure 1). The CRISPR-Cas9 plasmid to target the first possible CRISPR site in the CCR5 gene was assembled in the pX458 vector (also referred to as pSpCas9(BB)-2A-GFP; Addgene plasmid #48138) using the standard assembly protocol (Ran *et al.*, 2013a). The necessary annealing primers that produce the insertion sequence encoding the target-complementary sequence of the sgRNA in the assembled CRISPR plasmid were designed manually (Figure 2). The assembled CRISPR-Cas9 mediates a no-overhang (blunt) double strand break between the 24^th^ and 25^th^ nucleotides downstream from the CCR5 start codon (Figure 1).

### Cellular transfections

The plasmids encoding the TALEN right and left arms were simultaneously co-transfected with the pRGS-CR reporter plasmid at a ratio of 1:1:2 in a total of 2 μg of DNA and the CRISPR-Cas9-encoding plasmid was transfected in a total of 3 μg of DNA. For DNA transfection in both cases, Lipofectamine 2000^TM^ was used according to the manufacturer’s instructions and HEK293T cells were co-transfected at confluence (4x10^5^ cells/well) in six-well plates (BD Falcon, Corning).

### Flow cytometry (FC) and fluorescence activated cell sorting (FACS)

Flow cytometry was done using a BD Accuri C6 flow cytometer 24, 48 and 72 h after transfection to determine the highest proportion of GFP^+^ transfected HEK293T cells in the CRISPR-Cas9 transfections. This same approach was previously used to show that the interval with the highest proportion of RFP^+^/GFP^+^ cells in TALEN+pRGS-CR transfections was 72 h after transfection (Kim *et al.*, 2011; Nerys-Junior *et al.*, 2014) and this interval was used in cell sorting of TALEN+pRGS-CR transfections. For CRISPR-Cas9-transfected HEK293T cells, the best interval (highest proportion of GFP^+^ cells) was 48 h post-transfection. Cell sorting was done with a MoFlo^TM^ flow cytometer (Dako Cytomation, Beckman Coulter, Brea, CA, USA) to isolate HEK293T cells with the desired phenotype (RFP^+^/GFP^+^ for TALEN+pRGS-CR transfections and GFP^+^ for CRISPR-Cas9 transfections).

### Genomic extraction and DNA cloning

HEK293T cells were grown for five days after flow cytometric analysis and cell sorting. Genomic DNA was extracted from HEK293T cells using a QIAamp DNA blood mini kit (Qiagen, Hilden, Germany) and the CCR5 gene was amplified using the primers 5’-TGGAGGGCAACTAAATACATTCTAGG-3’ (forward) and 5’-CAGGTACCTATCGATTGTCAGGAGGA-3’ (reverse) with the following cycle conditions: 95 °C for 5 min in the pre-PCR phase, followed by 38 cycles of 95 °C for 30 s, 55 °C for 30 s and 72 °C for 1 min, with a final extension (post-PCR phase) of 72 °C for 7 min. The 445 bp amplicon, which included 200 bp up and downstream from the TALEN target site, and also 100 bp upstream and 300 bp downstream from the CRISPR-Cas9 site, was cloned using a pGEM^®^-T Easy Vector (Promega, Madison, WI, USA) and transformed in *E. coli* JM109 (Promega) competent cells. White positive colonies were screened using a Luria broth (LB) medium plate containing ampicillin (50 μg/mL), 5-bromo-4-chloro-3-indolyl-β-D-galactopyranoside (X-Gal) and isopropyl β-D-1-thiogalactopyranoside (IPTG). Subsequent comparison allowed distinction between sorted and unsorted white colonies.

### DNA sequencing

White colonies were sequenced using an ABI BigDye Terminator sequencing kit (Applied Biosystems, Carlsbad, CA, US) on an Applied Biosystems 3130 Genetic Analyzer. Genomic DNA extracted from non-transfected HEK293T cells was used as a wild-type reference that was validated based on the wild-type CCR5 genetic sequence from GeneBank (National Center for Biotechnology Information - NCBI, Bethesda MD, USA). All sequences were aligned using SeqMan software v8.1.2 (DNAStar, Madison, WI, USA).

## Results

**Figure 3:**
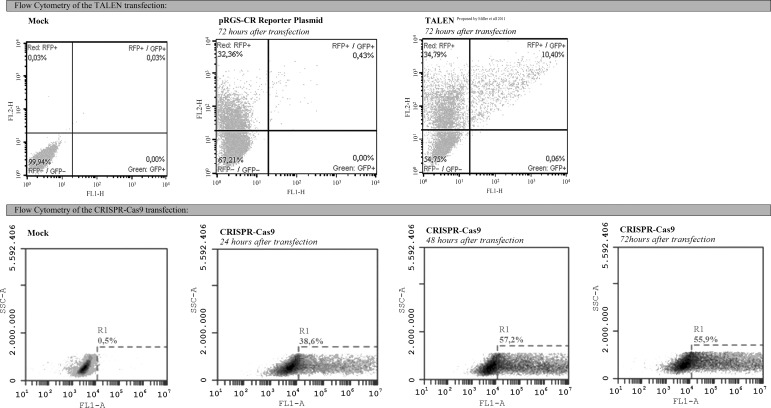
Flow cytometry 24, 48, and 72 h after CRISPR-Cas9 transfection and 72 h after TALEN transfection. Our previous work and other reports showed that the highest proportion of RFP^+^/GFP^+^ cells was obtained 72 h after TALEN transfection. The frequency of GFP^+^ cells in CRISPR-Cas9 transfections was compared with a mock transfection without DNA. This same mock transfection was used to measure the frequency of RFP^+^/GFP^+^ cells in TALEN transfections. The highest proportion of GFP^+^ cells (57.2%) was obtained 48 h after CRISPR-Cas9 transfection.

Fluorescence measurements by flow cytometry showed that GFP^+^ cells were most abundant in CRISPR-Cas9-transfected cells 48 h after transfection (Figure 3). Whereas Miller’s TALEN transfections resulted in ~10% of RFP^+^/GFP^+^ cells (Nerys-Junior *et al.*, 2014), CRISPR-Cas9 transfections resulted in 57.2% of GFP^+^ cells (Figure 3). The transfections were repeated 10 times in the same conditions and in all cases the proportion of gated cells showed no more than 2% variability.

Although CRISPR-Cas9 transfections were ~47% more efficient than TALEN transfections in generating GFP^+^ cells, as indicated by flow cytometric analysis, locus modifications still need to be evaluated by sequencing to show direct nucleotide In-Del alterations. For this, RFP^+^/GFP^+^ cells were sorted 72 h after TALEN transfections and GFP^+^ cells were sorted 48 h after CRISPR-Cas9 transfections. The sorted cells were cultured for five days in one well each of a 6-well plate, at which point they reached 80% confluence. The genomic DNA of both groups of cells was subsequently extracted for PCR. The resulting 445 bp amplicon containing TALEN and CRISPR-Cas9 sites was cloned into a pGEM^®^-T Easy Vector (Promega) and transformed in *E. coli* JM109 (Promega) competent cells that were then plated on an ampicillin/X-Gal/IPTG plate. A portion of cells was separated before cell sorting for subsequent extraction of genomic DNA and the amplicon containing TALEN and CRISPR-Cas9 target cloned into a pGEM^®^-T Easy Vector for subsequent comparison between sorted and unsorted cells.

**Figure 4:**
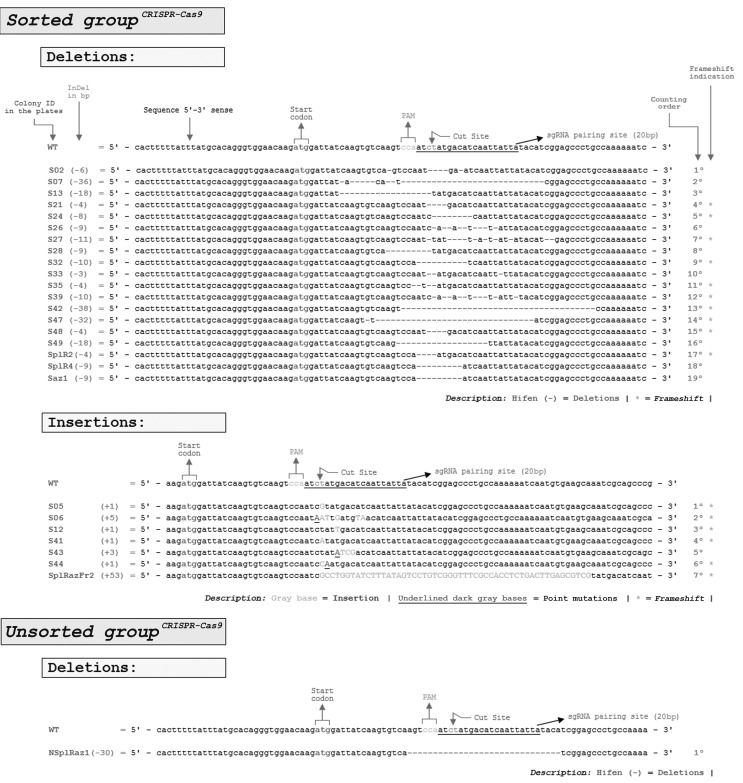
Genomic editions identified in the CRISPR-Cas9 transfections. For both sorted and unsorted groups 41 *E. coli* JM109 white colonies were sequenced by Sanger sequencing. Whereas only one colony was edited in the unsorted group, 26 colonies were edited in the sorted group, indicating 26-fold more gene editions in the sorted group compared to the unsorted group. While the only identified colony in the unsorted group was a 30-bp deletion, in the sorted group 73.1% of the genomic editing (19 colonies) consisted of deletions and 26.9% (7 colonies) consisted of insertions. In the sorted group, approximately two-thirds of the editing generated a frameshift (16 of 26 editions), indicating random mutations.

After Sanger sequencing, the analysis of 41 white colonies obtained from unsorted cells revealed only one CRISPR-Cas9-edited colony that contained a 30 bp deletion; all the other 40 white colonies were wild-type. In contrast, of 41 white colonies obtained from sorted cells, 26 were found to be CRISPR-Cas9-edited colonies. Of these, 73.1% (19 colonies) involved 4-36 bp deletions and 26.9% (7 colonies) involved 1-53 bp insertions in the CRISPR-Cas9 cut site (Figure 4).

In unsorted CRISPR-Cas9 transfected cells, only 2.4% of the white colonies (0.73 colonies/30 colonies analyzed) showed editing compared to sorted CRISPR-Cas9 transfected cells in which 63.4% of the colonies were edited (19 colonies/30 colonies analyzed). Thus, target gene editing was ~26-fold greater in CRISPR-Cas9 sorted white colonies than in unsorted white colonies.

**Figure 5:**
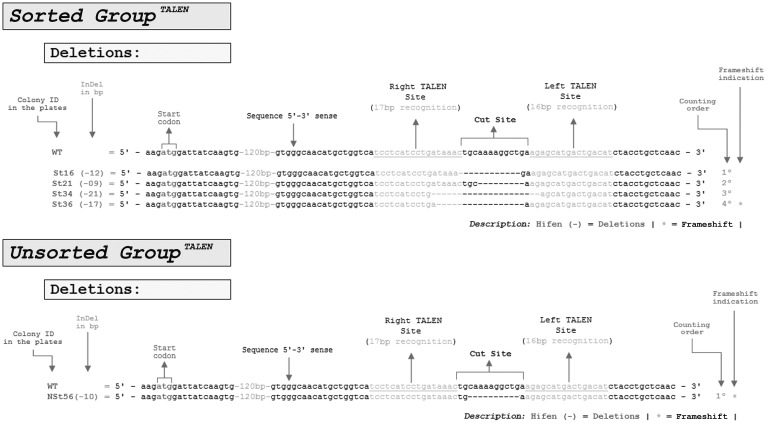
Genomic editing identified in TALEN transfections. For both sorted and unsorted groups 32 *E. coli* JM109 white colonies were sequenced by Sanger sequencing. Whereas only one colony was edited in the unsorted group, four colonies were edited in the sorted group, indicating four-fold more gene editing in the sorted group compared with the unsorted group. The only edited colony in the unsorted group contained a 10-bp deletion, while in the sorted group 100% of the genomic editing (four colonies) consisted of deletions and no insertions. Frameshift editing appeared to be random since around two-thirds of the deletions generated a frameshift.

Miller’s TALEN transfections resulted in one edited colony for every 30 colonies analyzed (3.3%, or 3.3 for every 100 analyzed) when no cell sorting was applied before genomic purification, and four edited colonies for every 30 analyzed (13.3%, 13.3 for every 100 analyzed) when genomic DNA extracted from sorted RFP^+^/GFP^+^ cells was analyzed five days after cell sorting (Nerys-Junior *et al.*, 2014). The sequencing of 32 white colonies in the sorted and unsorted groups yielded the same proportion as previously described. All the TALEN-edited colonies involved deletions ranging from 9 to 21 bp; no insertions were observed (Figure 5).

Table 1 summarizes the main findings of this investigation and shows that CRISPR-Cas9 was much better at editing the beginning of the CCR5 gene than the most efficient TALEN described for this same genetic site (Miller *et al.*, 2011; Nerys-Junior *et al.*, 2014).

**Table 1:** Summary of the differences between TALEN and CRISPR-Cas9 transfections. The percentage of gene knockouts from the total number of edited colonies (insertions and deletions) in CRISPR-Cas9 sorted cells was 61.5%.

	Cell sorting	Number of sequenced colonies	Number of edited colonies	% of edited colonies	Edited colonies/30 colonies sequenced	Improvement in efficiency between sorted and unsorted cases	Difference in efficiency between sorted TALEN and sorted CRISPR-Cas9	Deletions (%)	Insertions (%)	% of gene knock-out from the total number of edited colonies (deletions)	% of gene knock-out from the total number of edited colonies (insertions)	Deletion range (bp)	Insertion range (bp)
TALEN	Unsorted	32	1	3.1	0.9	~4 times	~5 times	100	0	100	0	10	None
	Sorted	32	4	12.5	3.8			100	0	25	0	9-21	None
CRISPR-Cas9	Unsorted	41	1	2.4	0.7	~26 times		100	0	0	0	30	None
	Sorted	41	26	63.4	19			73.1	26.9	52.6	85.7	4-36	1-53
								(n=19)	(n=7)				

## Discussion

TALEN and CRISPR-Cas9 transfections involve different reporter systems. Whereas TALEN requires the co-transfection of three plasmids (one for each TALEN arm and one for the pRGS-CR reporter system), CRISPR-Cas-9 assembled in the pX458 plasmid requires the transfection of only one plasmid that encodes the entire CRISPR-Cas9 system and the reporter system simultaneously.

Despite the disadvantage of TALEN compared to CRISPR-Cas9, when the need for co-transfections in TALEN experiments is required, in sorted RFP^+^/GFP^+^ TALEN-transfected cells TALEN production and action in the cell nucleus can be ensured, even though editing of the pRGS-CR reporter plasmid does not necessarily imply editing of the target gene (although this association is generally valid). On the other hand, not all editing of the pRGS-CR reporter plasmid will restore the GFP open reading frame and, in some cases, there may be target genome editing without internalization of the pRGS-CR reporter plasmid, or there may be target genome editing without editing of the internalized pRGS-CR reporter plasmid. Despite these limitations and uncertainties, for to assure TALEN entry and action within the target cells, the usage of the pRGS-CR reporter plasmid is currently the best option.

In experiments with CRISPR-Cas9 assembled in the pX458 vector, the on-board GFP reporter system allows easier transfections and ensures Cas9 production within the GFP^+^ cell, but does not indicate the anchorage of the sgRNA in Cas9 for the correct production, assembly and action of the CRISPR-Cas9 system within the cell nucleus. Nevertheless, the system mediated by the pX458 vector allows an extreme potential correlation between GFP^+^ cells and target genome editing.

The sorting of RFP^+^/GFP^+^ cells in TALEN transfections appears to be the only effective approach for ensuring TALEN production and action within the sorted cell nucleus. In the case of CRISPR-Cas9 assembly, the sorting of GFP^+^ cells in CRISPR-Cas9 transfections is a highly efficient procedure that ensures CRISPR-Cas9 system production within the sorted cell, therefore strongly indicating a potential target genome editing.

Our previous work (Nerys-Junior *et al.*, 2014) showed the same efficiency for TALEN transfection compared to that reported by Miller *et al.* (2011), and the efficiency observed here for CRISPR-Cas9 transfection was comparable to that of previous studies (Ran *et al.*, 2013a,b). However, direct comparison of the efficiencies of TALEN and CRISPR-Cas9 for the same genetic portion of the CCR5 gene under the same conditions is a new important finding that has a direct bearing on the development of CCR5 gene editing studies and new gene therapies in the CCR5 gene. In this context, the use of a shorter genetic site to evaluate the efficiency of gene editing by TALEN and CRISPR-Cas9 is an important consideration, especially because chromatin structure, transcription rate and DNA methylation of the chromosomal position of the target gene influence TALEN and CRISPR-Cas9 action equally within the cell nucleus. Thus, differences in efficiencies are directly related to the efficiencies of TALEN and CRISPR-Cas9 themselves and are locus-specific, i.e., they are not necessarily applicable to other genetic loci.

Off-target effects apparently did not affect our analysis of the efficiency of TALEN and CRISPR-Cas9 in editing the CCR5 gene. The off-target activity of the CRISPR-Cas9 system can be easily overcome by using a shorter (< 20 nucleotides) recognition portion of sgRNA that does not affect on-target CRISPR efficiency (Fu *et al.*, 2014).

In our experimental conditions, unsorted and sorted TALEN transfections generated 3.3 and 13.3 edited colonies, respectively, for every 100 colonies analyzed. Thus, cell sorting in TALEN transfections using the pRGS-CR reporter plasmid generates four times more editions than in the unsorted TALEN group. In contrast, unsorted and sorted CRISPR-Cas9 transfections generated 2.4 and 63.4 edited colonies, respectively, for every 100 colonies analyzed, indicating that cell sorting in CRISPR-Cas9 transfections using the pSpCas9(BB)-2A-GFP plasmid (*pX458*) as backbone generated 26 times more editions than in the unsorted CRISPR-Cas9 group. Together, these findings indicate that CRISPR-Cas9 was 4.8 fold more efficient than TALEN in editing the beginning of the CCR5 gene (13.3 edited colonies/100 colonies for sorted TALEN transfections versus 64.4 edited colonies/100 colonies for sorted CRISPR-Cas9 transfections). Our results also show that it is only possible to detect differences in efficiency when untransfected cells are separated from the correctly transfected cells.

In conclusion, CRISPR-Cas9 was better than TALEN for editing the beginning of the CCR5 gene, especially when greater editing efficiency and a higher proportion of edited cells are required.
